# Haematological and serum biochemical reference values in Chinese water deer (*Hydropotes inermis*): a preliminary study

**DOI:** 10.1186/s12917-020-02601-2

**Published:** 2020-10-20

**Authors:** Dayi Nie, Jianfeng Gui, Na Zhao, Yi Lin, Haiming Tang, Feng Cai, Guoping Shen, Jiazhong Liu, Endi Zhang, Min Chen

**Affiliations:** 1 School of Life Sciences, Institute of Eco-Chongming, East China Normal University, 500 Dongchuan Rd, Shanghai, 200241 China; 2 Yangtze Delta Estuarine Wetland Ecosystem Observation and Research Station, Ministry of Education & Shanghai Science and Technology Committee, Shanghai, 202162 China; 3 Shanghai Zoo, 2381 Hongqiao Rd, Shanghai, 200335 China; 4 Shanghai Pudong New Area Forestry Station, 285 East Huaxia Rd, Shanghai, 201210 China; 5 Shanghai Songjiang District Forestry Station, 839 Yinze Rd, Shanghai, 201620 China; 6 Shanghai Songjiang District Agricultural Commission, 1 Yuanzhong Rd, Shanghai, 201620 China

**Keywords:** Chinese water deer (*Hydropotes inermis*), Heamatological and biochemical parameters, Sex, Geographic origin

## Abstract

**Background:**

A selection of haematological and serum biochemical profile was first presented from the 81 samples of Chinese water deer (*Hydropotes inermis*). The deer health assessment database was initially established, especially in relation to determining potential effects associated with diseases diagnosis.

**Results:**

Blood samples were analyzed for different haematological parameters viz. white blood cells (WBC), red blood cells (RBC), haemoglobin (HGB), packed-cell volume (PCV), platelet count (PLT), mean corpuscular haemoglobin (MCH), mean corpuscular haemoglobin concentration (MCHC), mean corpuscular volume (MCV), mean red blood cells distribution width coefficient of variation (RDW) and different hematological parameters viz. total protein (TP), albumin (ALB), globulin (GLB), albumin to globulin ratio (A/G), total bilirubin (TBIL), alkaline phosphatase (ALP), γ-glutamyl transferase (GGT), alanine aminotransferase (ALT), aspartate aminotransferase (AST), AST/ALT, creatinine, urea (BUN), uric acid, total cholesterol (TC), triglyceride, creatine kinase (CK), lactate dehydrogenase (LDH) and cortisol. The adult females had higher values than adult males in albumin, mean corpuscular volume, packed-cell volume, and hemoglobin content values. The deer from Shanghai had higher urea nitrogen values than those from Zhoushan.

**Conclusion:**

To our knowledge this is the first report about the haematological and serum biochemical parameters in Chinese water deer. We had initially established a profile of Chinese water deer on haematological and serum biochemical parameters based on 81 samples we had collected. The findings can serve as a primary reference for health monitoring and disease prevention in this species.

## Background

Blood haematological and biochemical parameter are critical for the disease diagnosis and health management for both wild and captive animals. Blood parameters reflect the health condition of animals, and serum biochemistry is an important tool for clinical assessment of wildlife and livestock [1–4]. Haematological and biochemical parameters in different species have their own suitable range. Recently, animal blood as indicators of pathology not only occurred in livestock, but also in wildlife management [5, 6]. As widely distributed herbivores, deer occupy an important role in ecosystem and bring economic benefit to humans [7, 8]. Deer are game animals and their venison is eaten in some places. The health of deer may affect local ecosystems and economies. A growing number of studies have used blood serum parameters in deer health monitoring. For example, Quist et al.,(1997) monitored the status of white-tailed deer (*Odocoileus virginianus*) infected with EHDV-2, an epidemic haemorrhagic disease, by blood lymphocyte changes; Pareja et al., (2018) found that trace elements in the blood affected the antioxidant capacity of adult Red deer (*Cervus elaphus*) [9, 10]. However, for most cervid species there is still a lack of blood reference intervals for haematological and biochemical and parameters [11–15].

As the only species in Hydropotinae subfamily among Cervidae, the Chinese water deer (*Hydropotes inermis*) is mainly distributed in East China and the Korean peninsula [16]. However, it is a vulnerable species in the IUCN Red List with sharply shrinking populations [17, 18]. Previously, only one Chinese water deer of unknown sex and age had blood parameters reported [19]. For conservation purpose, the deer has been reintroduced to Shanghai since 2007 [20]. With the population increasing, normal haematological and serum biochemical data from a substantial and representative sample of Chinese water deer would be valuable in health monitoring and disease diagnosis. The objective of this study was to determine the haematological and serum biochemical reference values in Chinese water deer and the correlation with sex and geographical origin.

## Methods

### Sample collection

This study was collected 81 blood samples of Chinese water deer. Among them 74 from (Punan Woodland (E121.319388, N30.972463; altitude of 4 m), Huaxia Park (E121.655429, N31.19864; altitude of 4 m) of Shanghai, and the other 7 from Zhoushan archipelago (E122.296243, N29.982484; altitude of 86 m) of Zhejiang. The climate in both places is subtropical monsoon. The deer’s body mass is between 6.6 kg and 19.5 kg. These deer are all adults (sexual maturity or more than 1 year). The deer were divided into four groups: adult males, adult females, Shanghai deer (From Huaxia Park and Punan Woodland) and Zhoushan deer (From Zhoushan archipelago farm) in Table 1. The deer from Shanghai were fed soybean meal once a day in the morning and ate grass freely which was growing on the ground. The deer from Zhoushan were fed soybean meal and some farmland green materials, such as sweet potato vines. Water was available ad libitum both in Shanghai and Zhoushan. The blood samples were collected before transfer. Managers used a net to capture the deer in January, March, April, September and December. No anesthetics were used. The eyes were covered to ensure that they remain calm. All deer used in this study were examined and considered clinically healthy by a veterinarian. They had normal fur color, body condition and activity. Approximately 2 ml of blood was collected by venipuncture of the hindquarter into 2.7 ml tubes containing EDTA (Becton Dickinson and Company, Devon, UK) and 5 ml empty serum collection tubes (Becton Dickinson and Company, Devon, UK) for haematology and serum biochemistry respectively. All tubes were transported in an ice box, and then analyzed haematology and serum biochemical at Shanghai Labway Clinical Laboratory Company within 24 h. The samples need to be centrifuged at 10 °C and 3000 rpm for 10 min before testing. Due to the precision of the instrument, some biochemical parameters were not detected. In addition, our samples were collected in 3 years (2014, 2015 and 2016), with more parameters were involved in the later years. MCHC, MCH, RDW, PLT and Cortisol were added to the measurement of parameters since 2015, CK and LDH were added in 2016 (Additional file 1).

**Table 1:** Four groups of Chinese Water deer about sex and Location

Group	Shanghai		Zhoushan	Total
	HX	PN		
Adult male	24	26	7	57
Adult female	18	6	0	24
Total	74		7	81

HX means Huaxia Park, PN means Punan Woodland

### Haematological analysis

Blood samples were analyzed for different haematological parameters viz. white blood cells (WBC), red blood cells (RBC), haemoglobin (HGB), packed-cell volume (PCV), platelet count (PLT), mean corpuscular haemoglobin (MCH), mean corpuscular haemoglobin concentration (MCHC), mean corpuscular volume (MCV), mean red blood cells Distribution width coefficient of variation (RDW) using Automated Hematology Analyzer (Sysmex XE-5000, SYSMEX, Japan).

### Serum biochemical analysis

Blood samples were analyzed for different haematological parameters viz. total protein (TP), albumin (ALB), globulin (GLB), albumin to globulin ratio (A/G), total bilirubin (TBIL), alkaline phosphatase (ALP), γ-glutamyl transferase (GGT), alanine aminotransferase (ALT), aspartate aminotransferase (AST), AST/ALT, Creatinine, urea (BUN),Uric acid, total cholesterol (TC), Triglyceride, creatine kinase (CK), lactate dehydrogenase (LDH) using Automatic Biochemical Analyzer (Cobas-c702, Roche, Switzerland) and Cortisolusing Automatic Biochemical Analyzer (Cobas 8000-e602, Roche, Switzerland). The method is electrochemilu minescene immunoassay.

### Statistical analysis

The data was tested by Shapiro-Wilk, the result showed *p* > 0.05. This means that the data is non-normally distribution. Therefore, the significant differences were investigated using Wilcoxon signed rank test, the linear relationship was calculated by Spearman correlation, and baseline values were represented by median and quartile. Statistical analyses were performed using R software (R 3.4.4) [21]. All *p*-values were corrected using the Bonferroni method. The table and figure were designed using Microsoft Excel 2016.

## Results

### Sex

The results of the analysis of differences between sexes are shown in Table 2. The mean corpuscular volume (MCV) (*p* = 0.01026), the packed cell volume (PCV) (*p* = 0.01647), the MCH (*p* = 0.0378) and the albumin (ALB) (*p* = 0.0486) in the females were significantly higher than these in the males (Table 1). The creatine kinase (CK) in the females was three times higher than that of the males. The PLT in the males was twice as high as that in the females.

**Table 2:** Median and quartile interval of haematological parameters of male and female in Chinese water deer

Parameter	Adult male			Adult female		
	N	Median	(Q1, Q3)	N	Median	(Q1, Q3)
WBC(10^9/L)	53	4.57	(2.37, 7.43)	22	3.89	(2.12, 77.80)
MCV (fL)	53	37.90	(36.35, 39.75)	22	40.55	(38.20, 49.83) *
RBC(10^12/L)	53	11.98	(10.59, 13.08)	22	13.33	(11.24, 14.19)
HGB(g/dL)	53	17.30	(14.80, 18.75)	22	19.75	(16.38, 20.73)
PCV(%)	53	44.30	(40.60, 49.65)	22	53.85	(49.08, 62.08) *
MCHC(g/dL)	48	37.85	(35.53, 39.93)	14	36.80	(35.85, 40.10)
MCH (pg)	48	14.15	(13.90, 14.70)	14	14.90	(14.28, 15.10) *
RDW(%)	48	33.30	(31.73, 35.2)	14	34.45	(32.23, 35.38)
PLT(10^9/L)	48	323.50	(213.50, 421.75)	14	172.00	(116.00, 291.50)
TC (mmol/L)	38	1.88	(1.40, 2.18)	18	1.55	(1.39, 1.73)
Triglyceride (mmol/L)	38	0.34	(0.25, 0.46)	18	0.59	(0.36, 1.35)
CK(U/L)	29	734.00	(398.50, 1147.50)	7	1539.00	(1153.00, 2269.00)
LDH(U/L)	29	761.00	(563.00, 1206.00)	7	1113.00	(916.00, 1788.00)
GGT(U/L)	57	229.00	(172.50, 312.50)	24	213.00	(166.25, 272.00)
Cortisol (nmol/L)	48	205.00	(149.25, 270.25)	13	241.00	(154.50, 363.00)
BUN (mmol/L)	56	20.60	(15.73, 25.33)	24	16.90	(14.93, 19.23)
Creatinine (umol/L)	57	91.00	(74.00, 102.00)	24	90.00	(68.00, 116.75)
Uric acid (umol/L)	56	3.50	(1.00, 12.00)	18	4.50	(1.00, 12.00)
TP(g/L)	57	73.00	(67.00, 82.00)	24	71.00	(66.25, 74.00)
ALB(U/L)	57	33.00	(26.50, 36.00)	24	37.00	(33.00, 39.00)*
GLB(U/L)	57	42.00	(31.50, 51.50)	24	33.00	(29.25, 37.00)
A/G(%)	57	0.76	(0.52, 1.14)	24	1.11	(0.85, 1.31)
TBIL (umol/L)	57	1.80	(0.70, 2.70)	24	1.35	(0.90, 3.03)
ALT(U/L)	57	20.00	(16.00, 26.00)	24	22.50	(18.25, 28.75)
AST(U/L)	57	89.00	(67.00, 132.50)	24	80.00	(63.25, 126.00)
AST/ALT	57	4.50	(3.59, 6.25)	24	3.81	(2.56, 5.16)
ALP(U/L)	57	72.00	(57.00, 104.50)	24	66.50	(45.75, 103.00)

a. Haematological parameters include white blood cells (WBC), red blood cells (RBC), hemoglobin (HGB), packed-cell volume (PCV), platelet count (PLT), mean corpuscular haemoglobin (MCH), mean corpuscular haemoglobin concentration (MCHC),mean corpuscular volume (MCV), mean red blood cells Distribution width coefficient of variation (RDW). b. Biochemical parameters include total protein (TP), albumin (ALB), globulin (GLB), albumin to globulin ratio (A/G), total bilirubin (TBIL), alkaline phosphatase (ALP), γ-glutamyl transferase (GGT), alanine aminotransferase (ALT), aspartate aminotransferase (AST), AST/ALT, blood urea nitrogen (BUN), total cholesterol (TC), creatine kinase (CK), lactate dehydrogenase (LDH)

*: *p* < 0.05; **: *p* < 0.01; ***: *p* < 0.001

### Location

The only value showed significant differences between the deer from Shanghai and Zhoushan was the BUN, which in the deer from Shanghai was extremely higher (*p* = 0.00432) (Table 3).

**Table 3:** Median and quartile interval of haematological parameters of different location in Chinese water deer

Parameter	Shanghai			Zhoushan		
	N	Median	(Q1, Q3)	N	Median	(Q1, Q3)
WBC(10^9/L)	69	4.52	(2.19, 8.78)	6	3.37	(2.30, 5.00)
MCV (fL)	69	38.40	(36.0, 40.85)	6	39.00	(37.00, 39.70)
RBC(10^12/L)	69	12.27	(10.64, 13.35)	6	12.66	(11.84, 13.60)
HGB(g/dL)	69	17.80	(14.90, 19.56)	6	18.65	(17.36, 19.75)
PCV(%)	69	46.10	(41.10, 53.55)	6	48.05	(45.80, 53.83)
MCHC(g/dL)	56	37.40	(35.50, 40.00)	6	37.50	(36.70, 39.88)
MCH (pg)	56	14.20	(13.90, 14.80)	6	14.65	(14.45, 14.85)
RDW(%)	56	33.55	(31.73, 35.30)	6	33.15	(32.48, 33.60)
PLT(10^9/L)	56	307.50	(192.00, 417.75)	6	218.00	(137.00, 257.00)
TC (mmol/L)	49	1.67	(1.40, 2.16)	7	1.81	(1.40, 2.10)
Triglyceride (mmol/L)	49	0.37	(0.29, 0.60)	7	0.25	(0.10, 0.46)
CK(U/L)	29	967.00	(427.00, 1509.00)	7	888.00	(371.00, 1078.00)
LDH(U/L)	29	872.00	(721.00, 1434.50)	7	556.00	(542.00, 881.00)
GGT(U/L)	74	233.00	(171.50, 303.50)	7	203.00	(102.00, 226.00)
Cortisol (nmol/L)	54	217.50	(149.75, 273.25)	7	175.00	(123.00, 279.00)
BUN (mmol/L)	73	19.30	(16.70, 24.25)	7	11.70	(10.10, 13.70))**
Creatinine (umol/L)	74	91.00	(73.75, 105.00)	7	100.00	(74.00, 112.00)
Uric acid (umol/L)	67	5.00	(1.00, 12.00)	7	1.00	(1.00, 2.00)
TP(g/L)	74	71.00	(67.00, 79.50)	7	72.00	(67.00, 77.00)
ALB(U/L)	74	34.00	(27.00, 37.25)	7	35.00	(32.00, 35.00)
GLB(U/L)	74	37.00	(31.00, 50.25)	7	42.00	(31.00, 42.00)
A/G(%)	74	0.96	(0.53, 1.21)	7	0.84	(0.76, 1.11)
TBIL (umol/L)	72	1.40	(0.73, 2.80)	7	2.60	(2.20, 2.70)
ALT(U/L)	74	21.00	(16.75, 26.00)	7	20.00	(18.00, 27.00)
AST(U/L)	74	89.50	(66.00, 129.75)	7	74.00	(61.00, 79.00)
AST/ALT	74	4.33	(3.36, 6.14)	7	3.70	(3.20, 4.89)
ALP(U/L)	74	72.50	(53.00, 104.50)	7	58.00	(35.00, 74.00)

a. Haematological parameters include white blood cells (WBC), red blood cells (RBC), hemoglobin (HGB), packed-cell volume (PCV), platelet count (PLT), mean corpuscular haemoglobin (MCH), mean corpuscular haemoglobin concentration (MCHC),mean corpuscular volume (MCV), mean red blood cells Distribution width coefficient of variation (RDW). b. Biochemical parameters include total protein (TP), albumin (ALB), globulin (GLB), albumin to globulin ratio (A/G), total bilirubin (TBIL), alkaline phosphatase (ALP), γ-glutamyl transferase (GGT), alanine aminotransferase (ALT), aspartate aminotransferase (AST), AST/ALT, blood urea nitrogen (BUN), total cholesterol (TC), creatine kinase (CK), lactate dehydrogenase (LDH)

*: *p* < 0.05; **: *p* < 0.01; ***: *p* < 0.001

### Age and body condition

Among 27 indicators, there were no significant differences between the over 1 year and the under 1 year younger. Considering that the deer samples under 1-year-old have been sexually mature, 81 individuals were all considered as adults. The correlation between weight-related indicators and weight is shown in Table 4.In males, platelets count (PLT) (ρ = 0.528, *p* = 0.00069) and blood urea nitrogen (BUN) (ρ = 0.452, *p* = 0.00281) have a positive correlation with body mass, otherwise mean corpuscular haemoglobin (MCH) (ρ = − 0.583, *p* = 0.00008) showed a significant negative correlation with body mass. (Table 4).

**Table 4:** Correlation test of Chinese water deer weight and parameter value

Parameter	T		M		F	
	ρ	*p*-value	ρ	*p*-value	ρ	*p*-value
MCH (pg)	−0.234	0.40200	−0.583	0.00008 ***	0.213	2.79600
MCV (fL)	0.092	2.60400	−0.060	4.01400	− 0.364	0.57000
PCV (%)	−0.020	5.17800	−0.188	1.06200	−0.013	5.72400
PLT (10^9/L)	0.104	2.53200	0.528	0.00069 ***	−0.478	0.50400
ALB (U/L)	−0.029	4.79400	−0.223	0.57000	0.185	2.32800
BUN (mmol/L)	0.273	0.08400	0.452	0.00281 **	0.145	3.0

a.ρ is the Spearman correlation coefficient. b. T mean the total, M mean the male, F mean female.mean corpuscular haemoglobin (MCH), mean corpuscular volume (MCV), packed-cell volume (PCV), platelet count (PLT), albumin (ALB), blood urea nitrogen (BUN)

*: *p* < 0.05; **: *p* < 0.01; ***: *p* < 0.001

## Discussion

To our knowledge this is the first report about the haematological and serum biochemical parameters in Chinese water deer. This study is a comprehensive survey on the 81 samples of Chinese water deer so far examined. The data has been used to calculate reference values, and to analyze them by sex and location.

**Fig. 1:**
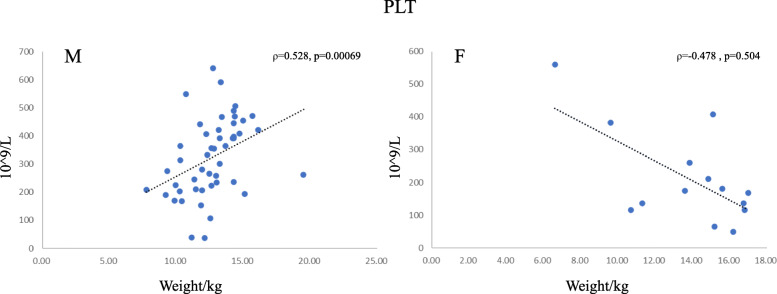
Spearman correlation coefficient between platelet count and body weight

The PLT in the males were much higher than that in the females without significant difference. However Parmar et al. (2017) found opposite result in Mehsana Goat (*Capra hircus*), mild higher in female goats,and slightly increased with age, which in the over 1 year’s were higher than in the under 1 year’s without explain [22] (Additional file 2). While the PLT values found in the juveniles are significantly higher than the adults for mouflon (*Ovis ammon*), Persian fallow deer (*Dama mesopotamica*) and Red deer, which might contribute to stronger haematopoietic capacity in juveniles [23–25]. We found the PLT and body mass showed significant positive correlation in the males (ρ = 0.528, *p* = 0.00012), and negative correlation in the females (ρ = − 0.478, *p* = 0.084) in Fig. 1. This may be the reason for males exhibited much higher PLT values than the females.

In this study, CK was found in myocardium and skeletal muscle and sensitive to muscle damage [26]. It exhibited much higher in the female Chinese water deer, but it had found the opposite tendency in fallow deer (*Dama dama L.*) and rusa deer (*Rusa timorensis*) [15, 27]. The CK value was found increasing rapidly when physical capture instead of chemical capture in red deer [13, 28]. Compared to fallow deer and rusa deer, the stress response of female was stronger in Chinese water deer [15, 27]. In addition, CK, lactate dehydrogenase (LDH), aspartate aminotransferase (AST) and alanine aminotransferase (ALT) were raised when transportation than capture, might be caused by increased stress for mouflon [23]. Beside CK, LDH, AST, and ALT all showed higher in female.

In this study, the ALP value in female Chinese water deer was only half of that in rusa deer ALP activity in blood is associated with the osteoblastic activity and also an index of skeletal and antler growth [15]. The ALP value was lower in Chinese water deer, but higher in rusa deer, and the gap was especially bigger in males [15]. Rusa deer have to support antlers develop, but Chinese water deer do not. ALP activity is associated with the osteoblastic activity and also an index of skeletal and antler growth [15].

**Fig. 2:**
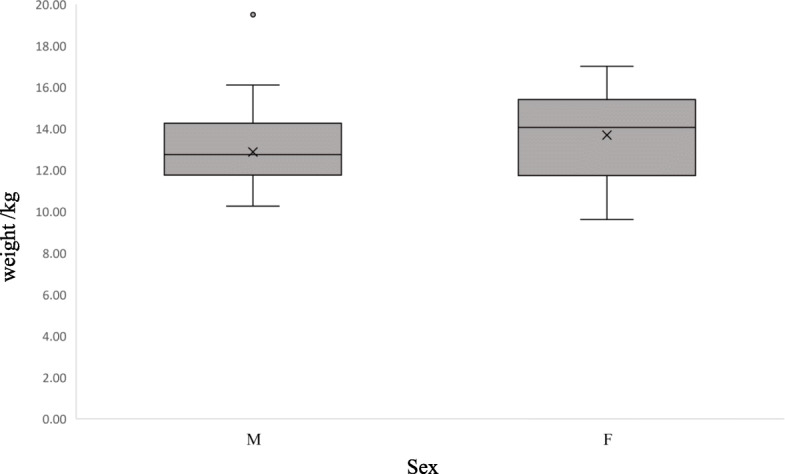
Weight of Chinese water deer in sex

There are several sex related differences significantly between the values for the males and the females. The higher for ALB, MCV, PCV and MCH obtained in the females (Table 2). The ALB, as a group of serum protein, may be influenced by nutrient condition in genus (*Odocoileus*) [29]. As was previously shown that our results found no correlation between body mass and ALB values Table 3). Higher body mass and protein intake may cause serum protein differences in bucks [27], but the females (14.05 kg) were heavier than males (12.74 kg) in the Chinese water deer we tested (Fig. 2). The result obtained by the previous study, the females 16.25 ± 1.77Kg) were heavier than the males (15.88 ± 1.44Kg) [16]. The studies on rusa deer, sika deer (*Cervus nippon yesoensis*) and grey-brocket deer (*Mazama gouazoubira*) found low MCV values in males as well, but there were no significant differences between the males and the females [30–32]. Besides cervid, other animals like cattle was found low MCV values in males [33]. However, in rusa deer, HO et al. (2018) found the MCV values for the females was significantly higher than the males [15]. PCV value for the females were higher than which for the males in grey-brocket deer, rusa deer, and fallow deer, respectively [24, 31, 32]. The high PCV values also found in goats (*Capra aegagrus hircus*) [34]. The MCV is the ratio of PCV to RBC. Although the RBC was no significant differences between two sexes, RBC in the females was slightly higher than those in the males. The differences observed in the MCV and PCV might be influenced by body conditions. The female Chinese water deer were heavier than the males (Fig. 2). Furthermore, the males in the Persian fallow deer had significantly higher MCH value [24]. We speculated that PCV and MCV increases were due to large oxygen consumption in females. Another possibility was that the tested females’ body mass was heavier than the males (Fig. 2). The females may have a higher oxygen demand, which would lead to an increase in the values for PCV and MCV.

**Fig. 3:**
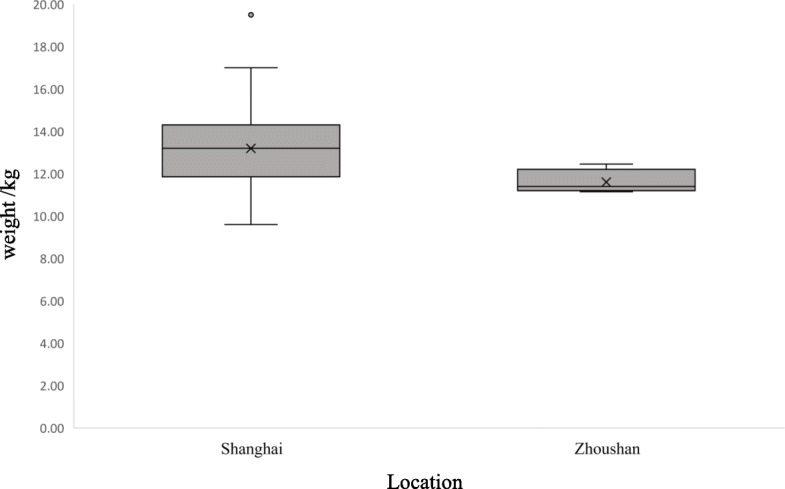
Weight of Chinese water deer in different locations

When investigating the difference between the Chinese water deer from two locations, the level of living condition and freedom level were considered. The study by Nina (2004) suggested that location might influence body condition via food resources and living space [27]. In our study, the BUN of the deer from Shanghai were significantly higher than the ones from Zhoushan. BUN was affected by high protein food catabolism and levels of rumen degradable proteins in a Red deer study [35]. We speculated that these differences in BUN were related to differences in body mass caused by nutrition levels (*p* = 0.012) (Fig. 3). The deer in Zhoushan feed on soybean meal and some farmland green materials, while the deer in Shanghai feed on soybean meal and ate grass freely, which may be the reason for the increase of the BUN value in Shanghai.

## Conclusions

We had initially established a profile of Chinese water deer on haematological and serum biochemical parameters based on 81 samples we had collected. The findings can serve as a reference for health monitoring and disease prevention in this species.

The sample size from Zhoushan in our data is very small, which is one of the shortcomings of this study. The reason was we only collected the blood samples during the capture and transfer process avoid extra disturbance to animals. The deer from the Zhoushan archipelago farm were rarely transferred, on the contrary, the deer in Shanghai transferred more. Another shortcoming in this study is that our blood parameters reference does not have pathological samples for comparison. We only selected clinically healthy individuals to collect samples to establish a reference system. These two points should be considered in future research. For rare animals, the physiological indicators are important for us to understand their health condition, but it needs a long-term study: we will continue to samples under different physical conditions in the future to enlarge and enhance the database.

## Supplementary information

**Additional file 1.** Detail information of measured parameters for the Chinese water deer.

**Additional file 2.** The parameter values of other animals from literatures in the discussion.

## Data Availability

The datasets analysed or generated during this study are available from the corresponding authors on reasonable request.

## Abbreviations

ALB: Albumin
ALP: Alkaline phosphatase
AST: Aspartate aminotransferase
ALT: Alanine aminotransferase
BUN: Blood urea nitrogen
CK: Creatine kinase
LDH: Lactate dehydrogenase
MCH: Mean corpuscular haemoglobin
MCV: Mean corpuscular volume
PLT: Platelets
PCV: Packed cell volume
